# Vulnerabilities in Older Patients when Cancer Treatment is Initiated: Does a Cognitive Impairment Impact the Two-Year Survival?

**DOI:** 10.1371/journal.pone.0159734

**Published:** 2016-08-01

**Authors:** Yves Libert, Stéphanie Dubruille, Cindy Borghgraef, Anne-Marie Etienne, Isabelle Merckaert, Marianne Paesmans, Christine Reynaert, Myriam Roos, Jean-Louis Slachmuylder, Sandrine Vandenbossche, Dominique Bron, Darius Razavi

**Affiliations:** 1 Université Libre de Bruxelles, Faculté des Sciences Psychologiques et de l’Éducation, Brussels, Belgium; 2 Université Libre de Bruxelles, Institut Jules Bordet, Brussels, Belgium; 3 Université de Liège, Faculté des Sciences psychologiques et de l’Education, Liège, Belgium; 4 Université Catholique de Louvain, Service de Médecine Psychosomatique, Cliniques Universitaires de Mont-Godinne, Yvoir, Belgium; University of Kentucky, UNITED STATES

## Abstract

**Introduction:**

Dementia is a known predictor of shorter survival times in older cancer patients. However, no empirical evidence is available to determine how much a cognitive impairment shortens survival in older patients when cancer treatment is initiated.

**Purpose:**

To longitudinally investigate how much a cognitive impairment detected at the initiation of cancer treatment influences survival of older patients during a two-year follow-up duration and to compare the predictive value of a cognitive impairment on patients survival with the predictive value of other vulnerabilities associated with older age.

**Methods:**

Three hundred and fifty-seven consecutive patients (≥65 years old) admitted for breast, prostate, or colorectal cancer surgeries were prospectively recruited. A cognitive impairment was assessed with the Montreal Cognitive Assessment (MoCA<26). Socio-demographic, disease-related, and geriatric vulnerabilities were assessed using validated tools. Univariate and subsequent multivariate Cox proportional hazards models stratified for diagnosis (breast/prostate cancer *versus* colorectal cancer) and disease status (metastatic *versus* non-metastatic) were used.

**Results:**

A cognitive impairment was detected in 46% (n = 163) of patients. Survival was significantly influenced by a cognitive impairment (HR = 6.13; 95% confidence interval [CI] = 2.07–18.09; p = 0.001), a loss in instrumental autonomy (IADL ≤7) (HR = 3.06; 95% CI = 1.31–7.11; p = 0.009) and fatigue (Mob-T<5) (HR = 5.98; 95% CI = 2.47–14.44; p <0.001).

**Conclusions:**

During the two years following cancer treatment initiation, older patients with a cognitive impairment were up to six times more likely to die than patients without. Older patients should be screened for cognitive impairments at cancer treatment initiation to enable interventions to reduce morbidity and mortality. Further studies should address processes underlying the relationship between cognitive impairments and an increased risk of dying in older cancer patients.

## Introduction

When starting cancer treatment, identifying sociodemographic, disease-related, and geriatric frailties that could increase morbidity and mortality among older patients has been recommended[1]. Frailties such as dementia have been recognized as predictor of shortened survival[2–5]. Indirect evidence suggests that cognitive impairments are also predictors of shortened survival in older cancer patients[6]^-8^. Although a recent study showed that approximately one-half of elderly cancer patients present with signs of cognitive impairments at cancer treatment initiation[7], no empirical evidence is currently available regarding how much a cognitive impairment influences survival in these patients.

At cancer treatment initiation, cognitive impairments in older patients could potentially shorten survival because they are associated with biological, medical, psychological, and/or social vulnerabilities. At the biological level, the presence of cognitive impairments has been associated with biomarkers that are associated with reduced life-expectancy. These biomarkers indicate changes such as advanced cell senescence, increased inflammation, decreased hormonal level, DNA damage, oxidative stress, or decrease in telomere length[8–11]. Cognitive impairments have also been associated with various comorbidities (poor cardiovascular conditions, diabetes, anemia, hypertension, or vitamin D deficiency[12]) and unhealthy lifestyles (low levels of physical activity, smoking, or alcohol abuse[11,12]) that have been shown to be associated with shortened life-expectancies in older people.

At the medical level, as it has been reported for dementia, cognitive impairments may be associated with an advanced stage of cancer at diagnosis[13]. Cognitive impairments may also be risk factors for delirium occurring during cancer treatments, which is a complication that has been recognized as a risk factor for a shorter life-expectancy among older patients[14]. Finally, cognitive impairments could be risk factors for an adjuvant under- or over-treatment of older patients. On one hand, not giving chemotherapy to some patients because of cognitive impairments may prevent them from potential remission. However, giving chemotherapy to patients with cognitive impairments may result in severe side effects that ultimately lead to life-threatening adverse events when these impairments are markers of underlying frailty or deficit in physiological function.

At the psychological level, the presence of cognitive impairments has been associated with characteristics recognized as risk factors for reduced life expectancies of older people in general and of older cancer patients in particular. These include anxiety, depression, distress, fatigue, low cognitive reserve, or neuropsychological disorders[15]. Finally, at the social level, cognitive impairments have been associated with low education levels and social isolation[16] that have been recognized as risk factors for reduced life expectancies in older people. Moreover, cognitive impairments may impair the abilities of older cancer patients to remember and implement recommendations from their relatives or health care professionals, specifically regarding cancer treatment (increase the risk of non-adherence) and the management of acute symptoms such as fever, nausea, or pain (increase the risk of life-threatening adverse events). These causes should be investigated as reasons why survival of older patients with cognitive impairments would be decreased.

Although previous studies identified various geriatric frailties that could shorten survival of older cancer patients, none examined precisely how much a cognitive impairment at the time of cancer treatment initiation could predict survival. Regarding cognitive frailties, these studies mainly assessed cognitive impairments based on patients’ medical records, with the brief mental status test or with the MMSE (Mini Mental State Examination). Although these assessment methods are useful to detect severe cognitive impairments in clinical practice, they are less sensitive to subtle impairments like criteria for a Mild Cognitive Impairment (MCI)[17–19] (i.e. concern regarding a change in cognition, impairment in one or more cognitive domains, preservation of independence in functional abilities and not demented). MCI is currently considered to be a transitional impairment between the cognitive changes of normal aging and the earliest clinical features of dementia[20]. However, it should be recalled at this level that among MCI patients, about 20% do not convert to dementia[21].

The Montreal Cognitive Assessment (MoCA) is a screening tool that has been specifically designed to assess MCI and dementia in first line specialty clinics[17]. However, in clinical and research state of the art, MoCA can be used to screen for cognitive impairments that may have other conditions that are ultimately diagnosed including delirium, long-standing cognitive impairment. These patients may not have the same risk of progression to dementia and it may be a disservice to patients and families if a diagnosis such as MCI or dementia is communicated. In order to reduce these risks, a comprehensive assessment including other health care professionals such as neuropsychologists and medical specialists in memory disorders should be conducted.

This study investigates prospectively, how much a cognitive impairment detected at the time of cancer treatment initiation predicts survival during a two-year follow-up duration in older patients, and to compare the predictive value of a cognitive impairment on patients survival with the predictive value of other vulnerabilities associated with older age. Patients were excluded if they were hospitalized in a palliative care unit and if they had dementia (i.e. loss of functional autonomy, disorientation, and severe memory problems). A two-year follow-up was performed, as this period was considered as the optimal time required for study deaths that could occur during surgery, adjuvant treatments, and recovery.

## Material and Methods

### Patients and setting

This longitudinal study was conducted in the medical-oncology department of a Belgian cancer center and was approved by the local ethics committee. All consecutive patients fulfilling the inclusion criteria were invited to participate and provided written informed consent. Patients were not compensated for their participation. To fulfill the inclusion criteria, patients had to be at least 65 years old, suffering from one of three types of cancer (breast, prostate, or colorectal) regardless of the disease stage, be hospitalized for cancer surgery, and be able to speak French. Patients were excluded if they were hospitalized in a palliative care unit, if they had a neoadjuvant therapy, if they had dementia (i.e. loss of functional autonomy, disorientation, and severe memory problems), if they were unable to adhere to the assessment schedule in this study for physical or psychological reasons. Recruited patients who were not recorded in the Belgium national population register (n = 4) were excluded. The first assessment (vulnerabilities assessments) was conducted in patients’ rooms during the first 48 hours after their admission or the second day following surgery if they could not be seen within the first 48 hours. The second assessment (evaluations of postoperative characteristics and main causes of death) was conducted 2 years later based on the patients’ medical records, on the Belgium national population register and on a phone contact with their general practitioner when necessary. The data used in the current longitudinal study have been partly used in a previously cross-sectional study addressing older cancer patients’ desire for a formal psychological help[7].

### Study and assessment procedure

#### The first assessment

First assessment lasted approximately one hour and was assisted by an independent investigator. Patients provided demographic information including age, gender, educational level, and living status. Physicians provided disease-related characteristics of patients including diagnosis, disease recurrence status, disease metastatic status, and intent to treat (adjuvant chemotherapy scheduled or not). The severity of surgery was measured by the Physiological and Operative Severity Scoring system for enumeration of Mortality and morbidity (POSSUM)[22].

Cognitive impairments were assessed using the Montreal Cognitive Assessment (MoCA)[17]. As systematically, the MMSE was realized before the MoCA, similar items such as the orientation and the attention tests were made only once. As the MMSE is well-known, only the MoCA will be described here.

The MoCA screening test requires respondents to answer questions, read instructions, and perform tasks with a writing instrument. The MoCA was developed to screen for a probable MCI. The MoCA is a one-page document that measures 8 cognitive domains: visuospatial/executive (5 points), naming (3 points), attention (6 points), language (3 points), abstraction (2 points), delayed recall (5 points), and orientation (6 points). Scores on the MoCA can range from 0 to 30. One point is added for an individual who has 12 years or fewer of formal education. A cut-off score of <26 is used to detect cognitive impairments. With this cut-off the MoCA has a sensitivity of 90% and a specificity of 87% to detect a probable MCI in older patients[17]. However, MoCA sensitivity and specificity have been found to vary tremendously in different clinical populations[23].

Finally, geriatric vulnerabilities of each patient were assessed using the following validated tools: Activities of Daily Living (ADL)[24], Instrumental Activities of Daily Living (IADL)[25], Timed up & Go test (TUG)[26], Falls during the last year (Falls), Mobility-Tiredness scale (Mob-T)[27], Mini Nutritional Assessment (MNA)[28], Hospital Anxiety and Depression Scale (HADS)[29], Geriatric Depression Scale in four items (GDS-4)[30], number of drugs (drugs)[31], Charlson Comorbidity Index (CCI)[32]. Each test used score cut-offs that were derived from the literature (ADL ≤5, IADL ≤7, TUG ≥15, Falls ≥2, Mob-T <5, MNA ≤23.5, HADS ≥13, GDS-4 ≥1, Drugs ≥5, CCI ≥2). Although the cut-off of the HADS is usually 11, the recommended cut-off score of 13 in the French version of the HADS was used as it gives 75% sensitivity and 75% specificity for screening for adjustment disorders and major depressive disorders taken together[33].

#### The second assessment

Medical records provided postoperative characteristics of patients including the length of postoperative stay and the short-term postoperative complications. Dates of deaths were extracted from the Belgium national population register allowing to ensure that all deaths were counted. Finally, main causes of death were extracted from the patients’ medical records and/or provided by the patients’ general practitioner. Length of postoperative stay was defined as the number of days spent in hospital after surgery. Postoperative complications were defined as any complication (≥ grade I) during the hospitalization following surgery.

### Statistical analyses

The study endpoint was overall survival measured from the Belgium national population register. Deaths occurring after 2 years of follow-up were not taken into consideration (observations were censored at 2 years). Univariate Cox regression analysis was performed in order to assess the relationships between each sociodemographic, disease-related, a cognitive impairment (based on the MoCA score), or other geriatric vulnerabilities and survival. Since this analysis was limited by the number of deaths observed after two years of follow-up (n = 24), extensive multivariate analyses could not be performed (it is recommended to have at least 10 events per covariate to be included in a model). However, in order to control for the impact of major disease-related covariates, subsequent multivariate Cox regression analyses that were stratified for diagnosis (breast/prostate *versus* colorectal) and disease status (metastatic *versus* non-metastatic) were performed in order to assess the relationship between a cognitive impairment or other geriatric vulnerabilities and survival (separate models for each geriatric vulnerability). These two stratifying medical variables were chosen because they may have a potential higher impact on survival than other assessed variables. Regarding diagnosis, we compared breast/prostate versus colorectal cancer because mortality rates of breast and prostate cancers are quite similar among older cancer patients and comparatively lower than mortality rates of colorectal cancer[34]. Stratified models were fitted only for covariates with univariate p values < 0.05. The effect of each characteristic on patient survival was expressed as hazard ratio (HR) with the corresponding 95% confidence interval (CI). In order to assess what could be other confounding factors for the prognostic role of a cognitive impairment on survival, associations between a cognitive impairment and sociodemographic, disease-related, and other geriatric vulnerabilities were assessed using appropriate parametric or non-parametric tests (Pearson and Spearman correlation coefficients, Student’s t-test, Mann-Whitney U test, and X2–test). Kaplan-Meier survival estimates were also generated to compare survival of patients with and without cognitive impairment. Statistical significance was defined as p <0.05. All statistical tests were two-sided. Statistical analyses were performed using SPSS software (IBM SPSS Statistics (v. 22); SPSS, Inc., Chicago, IL, USA).

## Results

### Patients’ recruitment, follow-up, and vulnerabilities

Of the 559 eligible patients, 89 refused to take part in the study and 113 inpatients were excluded because they did not answer the MoCA and decided to stop completing the questionnaire. The final sample consisted of 357 patients for whom 24 deaths were observed in the Belgium national population register. It should be noted that 30% of the recruited patients were assessed the second day following surgery. The sociodemographic, disease-related, a cognitive impairment, and other geriatric vulnerabilities of survivors and non-survivors at 2 years are listed in Tables 1 and 2.

**Table 1:** Sociodemographic and disease-related characteristics of older patients at the time of cancer treatment initiation, stratified for Survivors/Non-Survivors at 2 years (n = 357).

	**All (n = 357)**			**Survivors (n = 333)**			**Non-Survivors (n = 24)**		
	N		%	N		%	N		%
***Sociodemographic characteristics***									
**Age (years)**									
Mean		72			72			74	
SD		6			6			6	
**Gender**									
Men	112		31	102		31	10		42
Women	245		69	231		69	14		58
**Educational level**									
Junior high school or lower	212		59	196		59	16		67
High school graduation or higher degree	145		41	137		41	8		33
Living status*									
Alone	121		34	112		34	9		38
With partner, family, in nursing home or in institution	235		66	220		66	15		62
***Disease-related characteristics***									
**Diagnosis**									
Breast cancer	228		64	217		65	11		46
Prostate cancer	93		26	88		26	5		21
Colorectal cancer	36		10	28		9	8		33
**Disease recurrence**									
Initial cancer	296		83	284		85	12		50
Cancer relapse	61		17	49		15	12		50
**Disease status**									
Non-metastatic	316		89	307		92	9		38
Metastatic	41		11	26		8	15		62
**Intent to treat**									
Adjuvant chemotherapy scheduled	94		26	80		24	14		58
Adjuvant chemotherapy not scheduled	263		74	253		76	10		42
Severity of surgery**									
Low-Middle (I-II)	318		89	304		90	15		62
High-Very high (III-IV)	39		11	33		10	9		38
**Postoperative length (days)**									
Mean		5			5			8	
SD		6			5			7	
Postoperative complications***									
Yes	15		4	12		4	3		13
No	342		96	321		96	21		87
**Causes of death**									
Disease progression	-		-	-		-	20		83
Cardiovascular disease	-		-	-		-	3		13
Infection	-		-	-		-	1		4

*Percentages do not take missing data into account

**Assessed with the Possum scale

***Any medical complication reported in medical records; 2 patients had declared a delirium after surgery

**Table 2:** Cognitive Impairment (CI) and other geriatric vulnerabilities of older patients at the time of cancer treatment initiation, stratified for Survivors/Non-Survivors at 2 years (n = 357).

	All (n = 357)		Survivors (n = 333)		Non-Survivors (n = 24)	
	N	%	N	%	N	%
***Cognitive Impairment (CI)***						
Montreal Cognitive Assessment (MoCA)*						
Impaired	163	46	143	43	20	83
Not impaired	194	54	190	57	4	17
Other geriatric vulnerabilities**						
**Mini Mental State Examination (MMSE)**						
Vulnerable	47	13	45	14	2	8
Not vulnerable	310	87	288	86	22	92
**Activities of Daily Living (ADL)**						
Vulnerable	8	2	7	2	1	4
Not vulnerable	349	98	326	98	23	96
**Instrumental Activities of Daily Living (IADL)**						
Vulnerable	61	17	52	16	9	38
Not vulnerable	296	83	281	84	15	62
Time-up and Go test (TUG)***						
Vulnerable	9	3	8	3	1	5
Not vulnerable	248	97	325	97	23	95
Falls during the last year (Falls)***						
Vulnerable	24	7	21	6	2	8
Not vulnerable	333	93	312	94	22	92
**Mobility-tiredness scale (Mob-t)**						
Vulnerable	119	33	103	31	16	67
Not vulnerable	238	67	230	69	8	33
**Mini Nutritional Assessment (MNA)**						
Vulnerable	58	16	52	16	6	25
Not vulnerable	299	84	281	84	18	75
**Hospital Anxiety and Depression Scale (HADS)**						
Vulnerable	114	32	103	31	11	46
Not vulnerable	243	68	230	69	13	54
**Geriatric Depression Scale four items (GDS-4)**						
Vulnerable	216	61	200	60	16	67
Not vulnerable	141	39	133	40	8	33
**Number of drugs (Drugs)**						
Vulnerable	112	31	104	31	8	33
Not vulnerable	245	69	229	69	16	67
**Charlson Comorbidity Index (CCI)**						
Vulnerable	184	52	166	50	18	75
Not vulnerable	173	48	167	50	6	25

*The cut-off point of <26 is used to detect CI

**Each tool was scored on a dichotomous scale, based on individual cut-off points reported in the literature

***Percentages do not take missing data into account

### Patients’ cognitive impairments

A cognitive impairment was experienced by 46% of the recruited older patients according to the MoCA scores. The mean MoCA score was 25.4 (SD = 3.6) and the score values ranged from a minimum of 10 to a maximum of 30. It should be underlined that none (n = 0) of older cancer patients experienced a severe cognitive impairment (0–9 regarding MoCA scores), 9% (n = 30) experienced a moderate cognitive impairment (10–19 regarding MoCA scores) and 37% (n = 133) experienced a mild cognitive impairment (20–25 regarding MoCA scores). MoCA scores were associated with IADL (r = 0.161; p = 0.002), TUG (r = -0.211; p <0.001), Mob-t (r = 0.216; p <0.001), MMSE (r = 0.650; p <0.001), HADS (r = -0.194; p <0.001), number of drugs (r = -0.169; p = 0.001), age (r = -0.321; p <0.001), and a lower educational level (p <0.001). There was no significant difference between MoCA scores of patients assessed within the first 48 hours of their hospital stay (n = 252; 70%; M = 25.6; SD = 3.5) and those assessed the second day following surgery (n = 105; 30%; M = 24.8; SD = 3.8) (p = 0.063).

### Prediction of patients’ two-year survival

Characteristics associated with survival among older patients (univariate Cox regressions) are listed in Table 3. Among the sociodemographic and disease-related characteristics, the diagnosis (p <0.001), disease recurrence status (p <0.001), disease metastatic status (p <0.001), intent to treat (p = 0.001), severity of surgery (p <0.001), postoperative length (p = 0.016), and postoperative complications (p = 0.038) predicted survival. A cognitive impairment measured with a cut-off score (MoCA <26) (p = 0.001) and as a continuous variable (p = 0.017) predicted survival. The small number of deaths (n = 24) did not allow to see whether subgroups with severe versus milder cognitive impairment experience similar rates of mortality. Among other geriatric vulnerabilities, loss of autonomy (IADL ≤7) (p = 0.008), fatigue (Mob-T <5) (p = 0.001), and comorbidities (CCI >1) (p = 0.023) were predictive for survival.

**Table 3:** Associations between sociodemographic characteristics, disease-related characteristics, Cognitive Impairment (CI) and other geriatric vulnerabilities of older patients at the time of cancer treatment initiation, and two-year survival: Univariate Cox regressions.

	HR	95% CI	*P*
***Sociodemographic characteristics***			
Age	1.04	0.98 to 1.11	0.216
Men *vs*. women	1.61	0.72 to 3.62	0.250
Junior high school or lower *vs*. high school graduation or higher degree	1.38	0.59 to 3.23	0.457
Alone *vs*. with partner, family, in nursing home or in institution*	1.16	0.51 to 2.66	0.721
***Disease-related characteristics***			
Colon cancer *vs*. breast or prostate cancer	4.92	2.11 to 11.50	<0.001
Cancer recurrence *vs*.initial cancer	5.26	2.36 to 11.70	<0.001
Metastatic *vs*. non-metastatic	15.81	6.91 to 36.17	<0.001
Adjuvant chemotherapy scheduled *vs*. no adjuvant chemotherapy scheduled*	3.89	1.71 to 8.88	0.001
High-very high surgery *vs*. low-middle surgery**	5.26	2.30 to 12.02	<0.001
Postoperative length	1.05	1.01 to 1.09	0.016
Postoperative complications *vs*. non postoperative complications***	3.60	1.08 to 12.08	0.038
***Cognitive Impairment (CI)***			
Montreal Cognitive Assessment (MoCA<26)	6.26	2.14 to 18.31	0.001
Other geriatric vulnerabillities****			
Mini Mental State Examination (MMSE)	1.70	0.40 to 7.23	0.473
Activities of Daily Living (ADL)	1.86	0.25 to 13.75	0.545
Instrumental Activities of Daily Living (IADL)	3.05	1.34 to 6.97	0.008
Time-up and Go test (TUG)*	1.72	0.23 to 12.84	0.595
Falls during the last year (Falls)*	1.28	0.30 to 5.44	0.742
Mobility-tiredness scale (Mob-t)	4.20	1.80 to 9.81	0.001
Mini Nutritional Assessment (MNA)	1.78	0.71 to 4.49	0.221
Hospital Anxiety and Depression Scale (HADS)	1.82	0.82 to 4.06	0.144
Geriatric Depression Scale four items (GDS-4)	1.30	0.56 to 3.04	0.545
Number of drugs (Drugs)	1.09	0.47 to 2.55	0.842
Charlson Comorbidity Index (CCI)	2.92	1.16 to 7.34	0.023

*Percentages do not take missing data into account

**Assessed with the Possum scale

***Any medical complication reported in medical records

****Each tool was scored on a dichotomous scale, based on individual cut-off points reported in the literature

**Fig 1:**
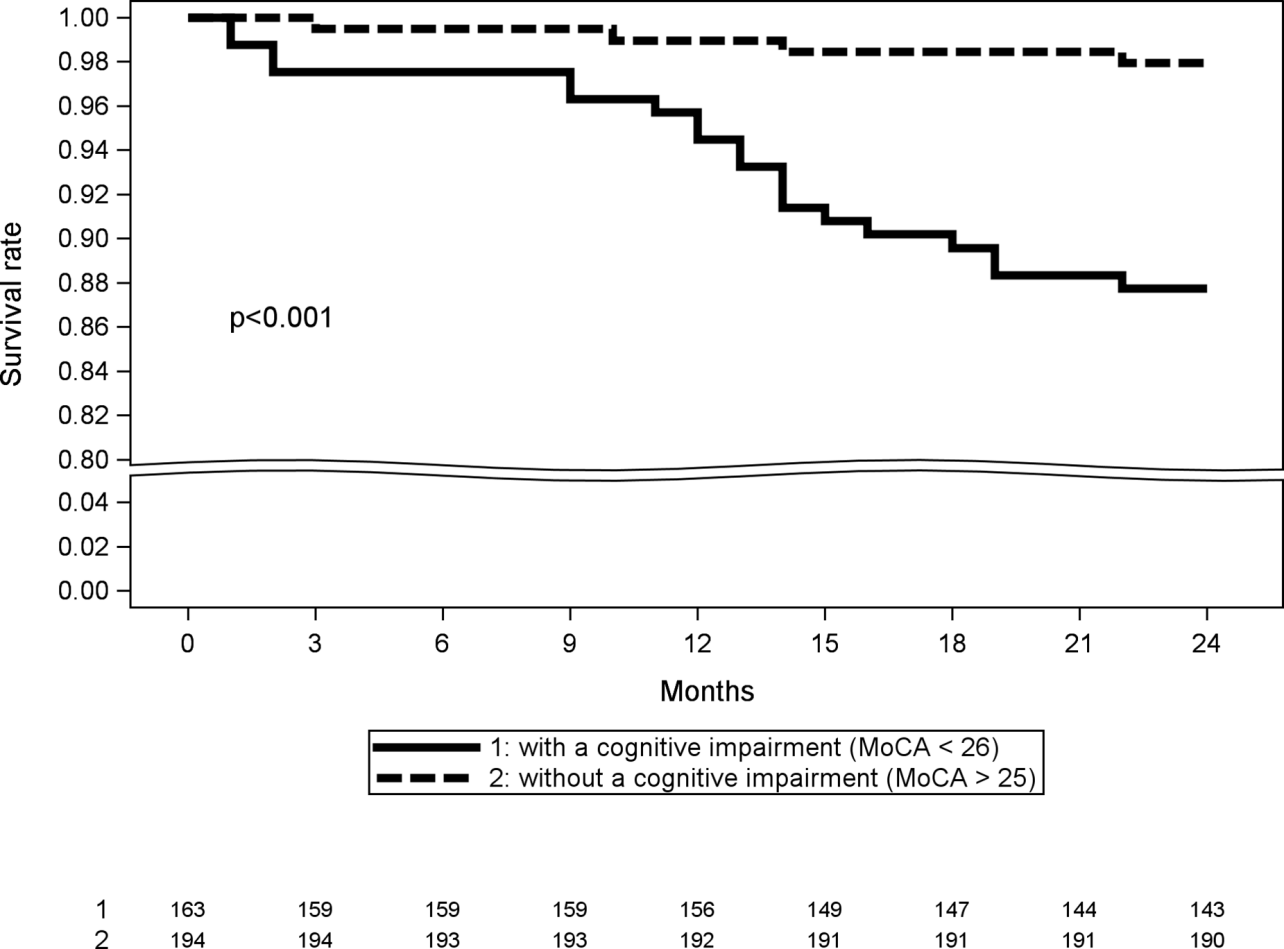
Kaplan-Meir overall survival estimates of two-year overall survival between older patients without a cognitive impairment (MoCA ≥26; n = 4) and patients with a cognitive impairment (MoCA <26; n = 20) when treatment for breast, prostate, or colorectal cancer is initiated.

Cognitive impairments and other geriatrics vulnerabilities associated with survival of older patients (subsequent multivariate Cox regressions stratified for diagnosis and disease metastatic status) are listed in Table 4. Overall survival was predicted by a cognitive impairment (MoCA <26) (HR = 6.13; 95% CI = 2.07–18.09; p = 0.001), loss of instrumental autonomy (IADL ≤7) (HR = 3.06; 95% CI; 1.31–7.11; p = 0.009) and fatigue (Mob-T <5) (HR = 5.98; 95% CI; 2.47–14.44; p <0.001). Fig 1 shows the Kaplan-Meier plot of survival between patients without a cognitive impairment (MoCA ≥26) and patients with a cognitive impairment (MoCA <26). Patients with a cognitive impairment had a higher mortality risk (p <0.001) and the majority of deaths occurred after one-year (62%). The majority of patients (83%) died due to a disease progression.

**Table 4:** Associations between Cognitive Impairment (CI) and other geriatric vulnerabilities of older patients at the time of cancer treatment initiation, and two-year survival*: Multivariate Cox regressions stratified for diagnosis and disease status**.

	HR	95% CI	*P*
***Cognitive Impairment (CI)***			
Montreal Cognitive Assessment (MoCA<26)	6.13	2.07 to 18.09	0.001
Other geriatric vulnerabilities***			
Instrumental Activities of Daily Living (IADL)	3.06	1.31 to 7.11	0.009
Mobility-tiredness scale (Mob-t)	5.98	2.47 to 14.44	<0.001
Charlson Comorbidity Index (CCI)	2.08	0.82 to 5.29	0.125

*Analysis was limited by the number of events we reached at two years (n = 24), therefore subsequent multivariate Cox regressions stratified for diagnosis and disease status were performed

**Non-metastatic breast/prostate cancer vs. metastatic breast/prostate cancer vs. metastatic colon cancer. No non-metastatic colorectal cancer died during the follow-up

***Each tool was scored on a dichotomous scale, based on individual cut-off points reported in the literature; MMSE, ADL,TUG, Falls, MNA, HADS, GDS-4, Drugs were not included in the multivariate analyses because they were not associated with two-year survival in univariate analyses

## Discussion

The first aim of this longitudinal study was to investigate how much a cognitive impairment assessed by the MoCA when cancer treatment is initiated and assessed by the MoCA, predicted survival in older cancer patients. A cognitive impairment was detected in 46% of the 337 recruited older patients. The total mortality rate for patients was 7% (n = 24) after two years. During the two years following surgery, when stratified by diagnosis (breast/prostate cancer *versus* colorectal cancer) and disease status (metastatic *versus* non-metastatic), older cancer patients with a cognitive impairment were up to six times more likely to die than patients without a cognitive impairment. It should be noted that the increased risk of death due to a cognitive impairment was almost identical in univariate analyses (HR = 6.26) and in analyses stratified for diagnosis and disease metastatic status (HR = 6.13).

The second aim of this study was to compare the predictive value of a cognitive impairment to that of other vulnerabilities associated with older age. Loss of instrumental autonomy and fatigue were the only other vulnerabilities predicting survival at two years. This confirms results of other studies[35,36]. It is important to note that a cognitive impairment had a predictive value similar to the one of fatigue and twice that of a loss of autonomy, and that both factors were weakly correlated with MoCA scores. Results of this study suggest that a cognitive impairment is an independent risk factor of death during 2 years of follow-up in older patients, regardless of the presence of other well-characterized medical or geriatric risk factors.

The relatively high prevalence of a cognitive impairment observed at baseline (46%) is consistent with results from other recent studies that noted the high rates of cognitive impairments in patients at treatment initiation[37]. These cognitive impairments may be due to aging and/or to the adverse biological effects of cancer itself, known as the “*cancerbrain*” concept, through increased inflammation, unregulated cytokines, or oxidative stresses[11,37]. It should be also noted that 30% of the recruited patients were assessed the second day following surgery. Some observed cognitive impairments could thus be induced by general anesthesia[14,38]. It should be also noted that 17% of the recruited patients were admitted for surgery for recurrent solid tumors. The observed cognitive impairments could also be partly due to long-term side-effects of previous cancer treatments such as chemotherapy[15]. Finally, it should be recalled at this level that if patients in this study were excluded if they had a diagnosis of dementia (i.e. loss of functional autonomy, disorientation, and severe memory problems), many studies have found under detection of dementia in clinical populations[39].

This study did not seek to investigate the processes by which cognitive impairments predicted a shortened survival. Nevertheless, only 4% of patients developed post-operative complications and 62% of observed deaths occurred after one-year, mainly due to disease progression (83%). From these observations, we can exclude that the majority of observed deaths were due to surgical complications or an adjuvant overtreatment of patients with frailties or compromised physiological function. Having a cognitive impairment at the initiation of cancer treatment seems to predict death for causes other than life-threatening surgical complications or adjuvant overtreatment of patients.

In this study, a cognitive impairment detected by the MMSE (<27) was not a significant predictor of survival in any of the analyses performed. There are two possible explanations that may account for these results. First, few patients were found to be vulnerable by the MMSE (<27) (13%), reducing the potential predictive power of impairments detected by this scale. Second, although the MMSE is useful to detect severe cognitive impairment in clinical settings, it is less sensitive to subtle impairments than the MoCA is[17–19].

If further studies confirm that a cognitive impairment at cancer treatment initiation predicts survival in older patients, we suggest that cognitive impairment should be screened in this population in order to appropriately implement four supportive interventions to reduce morbidity and mortality for cognitively vulnerable patients (MoCA <26). First, it should be recalled, of course, that any positive screening score should be confirmed by a comprehensive cognitive assessment realized by a neuropsychologist and/or a medical specialist in memory disorders. Second, if diagnosis of a cognitive impairment is confirmed, we suggest that physicians and other health care professionals inform older patients about that. Furthermore, specific interventions that are needed should be explained to them. Third, we suggest that physicians propose to repeat cognitive assessments at least one year following surgery and modify the course of the potential adjuvant treatments according to the evolution of impairments. Fourth, we suggest proposing support interventions to increase compliance among patients with cognitive impairments regarding cancer treatment and management of acute symptoms such as fever, nausea, or pain. Potentially useful strategies that could be considered to increase compliance among patients with cognitive impairments include comprehensive patient education, neuro-psychological consultations, medication review, intensive post-discharge follow-up, and home-based interventions[40,41]. Informal primary caregivers and family physicians should be included in these supportive interventions in order to maximize their usefulness and potential benefits.

To our knowledge, this is the first study aimed at investigating how much a cognitive impairment detected at the initiation of cancer treatment influences survival of older patients during a two-year follow-up duration and at comparing the predictive value of a cognitive impairment on patients’ survival with the predictive value of other vulnerabilities. The MoCA and other measures included in the geriatric assessment are valid measures and provide a comprehensive picture of relevant vulnerabilities in this specific population. The results of this study have important potential practical implications and are in the line with the growing literature on the importance of detecting cognitive impairments not only in older people with cancer, but in other medical contexts. Future prospective studies should assess how much cognitive impairments predict survival in others settings (i.e., within a follow-ups of 3, 4, and 5 years; among outpatients; among patients with unfavorable prognoses; or among frail patients), with other measures combining for example MoCA cut-off scores and other cognitive tests, with larger samples to improve the power of survival prediction. Prospective studies, should also assess whether subgroups with severe versus milder cognitive impairments experience similar rates of mortality. Moreover, future prospective studies should include an assessment of any adjuvant cancer treatment and assessments of patient compliance with medical recommendations in order to better understand the processes by which cognitive impairments could reduce survival in older cancer patients. Among these processes, prospective studies should also assess biomarkers of aging that are also associated with cognitive impairments and survival (i.e. cell senescence, inflammation, hormone levels, DNA damage, oxidative stress, or telomere length). Prospective studies should also include repeated measures of cognitive impairments in order to investigate the advancement of impairments and their potential impact on survival. Finally, prospective studies should investigate the potential benefits of supportive interventions based on cognitive impairments screening that aim to reduce morbidity and mortality of older cancer patients.

In conclusion, during the two years following cancer treatment initiation, older cancer patients with a cognitive impairment may have up to six times greater risk of dying than patients without. A cognitive impairment may be an indication of a patient’s biological, medical, psychological, and social vulnerabilities. Older patients should be screened for cognitive impairments at cancer treatment initiation in order to propose supportive interventions aiming to reduce morbidity and mortality. Further studies should address processes underlying the relationship between cognitive impairments and an increased risk of dying among older cancer patients.
